# GPCRdb in 2023: state-specific structure models using AlphaFold2 and new ligand resources

**DOI:** 10.1093/nar/gkac1013

**Published:** 2022-11-18

**Authors:** Gáspár Pándy-Szekeres, Jimmy Caroli, Alibek Mamyrbekov, Ali A Kermani, György M Keserű, Albert J Kooistra, David E Gloriam

**Affiliations:** Department of Drug Design and Pharmacology, University of Copenhagen, Universitetsparken 2, 2100 Copenhagen, Denmark; Medicinal Chemistry Research Group, Research Center for Natural Sciences, Budapest H-1117, Hungary; Department of Drug Design and Pharmacology, University of Copenhagen, Universitetsparken 2, 2100 Copenhagen, Denmark; Department of Drug Design and Pharmacology, University of Copenhagen, Universitetsparken 2, 2100 Copenhagen, Denmark; Department of Structural Biology, St. Jude Children's Research Hospital, Memphis, TN 38105, USA; Medicinal Chemistry Research Group, Research Center for Natural Sciences, Budapest H-1117, Hungary; Department of Drug Design and Pharmacology, University of Copenhagen, Universitetsparken 2, 2100 Copenhagen, Denmark; Department of Drug Design and Pharmacology, University of Copenhagen, Universitetsparken 2, 2100 Copenhagen, Denmark

## Abstract

G protein-coupled receptors (GPCRs) are physiologically abundant signaling hubs routing hundreds of extracellular signal substances and drugs into intracellular pathways. The GPCR database, GPCRdb supports >5000 interdisciplinary researchers every month with reference data, analysis, visualization, experiment design and dissemination. Here, we present our fifth major GPCRdb release setting out with an overview of the many resources for receptor sequences, structures, and ligands. This includes recently published additions of class D generic residue numbers, a comparative structure analysis tool to identify functional determinants, trees clustering GPCR structures by 3D conformation, and mutations stabilizing inactive/active states. We provide new state-specific structure models of all human non-olfactory GPCRs built using AlphaFold2-MultiState. We also provide a new resource of endogenous ligands along with a larger number of surrogate ligands with bioactivity, vendor, and physiochemical descriptor data. The one-stop-shop ligand resources integrate ligands/data from the ChEMBL, Guide to Pharmacology, PDSP Ki and PubChem database. The GPCRdb is available at https://gpcrdb.org.

## INTRODUCTION

G protein-coupled receptors (GPCRs) account for 799 (1) out of 20 383 (2) (4%) human genes and transduce the responses of two-thirds (342/515) of signaling substances (3). Their predominant role in physiology is mirrored in medicine, as 34% of marketed drugs target GPCRs (4). The GPCR database, GPCRdb serves ∼5000 researchers every month with reference data, analysis, visualization, experiment design and data deposition. GPCRdb is open access and open source, and compliant with the FAIR principles (5). In 2022, what was previously a G protein section of GPCRdb grew into a dedicated database, GproteinDb adding, e.g. a coupling atlas integrating major datasets (6), structure complexes with GPCRs, and interface interactions (7). Also ArrestinDb (8) and Biased Signaling Atlas (9) have been much expanded and are brought forward as separate resources covering complementary aspects of signal transduction while serving dedicated research communities and use cases.

GPCRdb has long provided state-of-the-art homology models from a unique multi-template pipeline. However, current advances call for a transition towards machine-learning based models taking advantage of recent breakthroughs. AlphaFold2 was, together with RoseTTA-fold, awarded the method and breakthrough of the year by *Nature* (10) and *Science* (11), respectively. While pre-generated AlphaFold2 models can be downloaded from the European Bioinformatics Institute (12), they have limitations of both general and GPCR-specific scope. Furthermore, AlphaFold models are provided for only one structural/functional state of each protein. To provide both inactive or active state GPCR models, a prediction protocol AlphaFold-MultiState (13) has been developed using two distinct sets of templates for the inactive and active states, respectively (13) based on state classifications from GPCRdb (14). However, the AlphaFold-MultiState models do not cover all GPCRs and were published without mention of long-term regular updates incorporating new templates (latest templates from June 2021).

Ligands of GPCRs span endogenous ligands, tool compounds, drug, and agents in clinical trials. The endogenous ligands are curated by experts coordinated by the authoritative Nomenclature Committee of the International Union of Basic and Clinical Pharmacology (NC-IUPHAR, https://www.guidetopharmacology.org/nomenclature.jsp). They are provided in the Guide to Pharmacology (GtP) database (15) along with a classification of a receptor's principal and secondary ligand (when multiple), binding affinity (pK_i_) and potency (pEC_50_) values. The largest source of tool compounds is ChEMBL (16), which contains over 206 000 GPCR ligands with binding (pK_i_, pK_d_) or functional (pEC_50_, (pIC_50_)) data. Information about drugs approved by the US Food and Drug Administration is available from DrugCentral and DrugBank. Another source of consistently determined binding affinities – for psychoactive endogenous, tool and drug ligands—is the K_i_ database from the Psychoactive Drug Screening Program (https://pdsp.unc.edu/databases/kidb.php). Furthermore, commercial availability at vendors and physicochemical properties are provided for nearly all ligands in the PubChem database (17). GPCRdb has previously integrated ChEMBL ligands and PubChem data (18,19).

In this article, we present an overview of all data and tool resource in our fifth major GPCRdb release, state-specific GPCR structure models (using AlphaFold2-MultiState and greatly expanded ligand resources (integrating all above ligand databases). These updates will increase GPCRdb's utility as a one-stop-shop for the GPCR community across basic research and drug discovery areas.

## METHODS

### Building state-specific GPCR structure models

Due to AlphaFold's limitation regarding lack of activation state specificity, we built 844 active or inactive state AlphaFold-MultiState models for 422 GPCRs in GPCRdb. We used structural templates published up until 4 July 2022 (published AlphaFold-MultiState models were based on templates until June 2021). The use of AlphaFold-MultiState removed the need for alternative backbone templates in our previously published GPCRdb's structure modelling pipeline (18,19). All generated models are based on the full-length wildtype sequence, except for AGRV1 for which partial models were generated since this receptor comprises 6306 amino acids which exceeded our computing capabilities.

### Building refined GPCR structures

Refined structures revert mutations to wildtype. Missing regions are filled in from an AlphaFold model based solely only the given experimental structure and fitted by superposition at junction sites.

### Updating of ligands and bioactivities

GPCR ligands and their biological activities were imported from the ChEMBL (v. 30) (16), Guide to Pharmacology (v. 2022.2) (15) and PDSP K_i_ databases (accessed 7 September 2022, https://pdsp.unc.edu/databases/kidb.php), as in (19). Ligand physicochemical properties and commercial availability data were retrieved from the PubChem database (17), as in (18). Information about FDA approval of drugs was imported from DrugBank. To enable data integration and links to external drug (DrugBank, and DrugCentral) and ligand databases (ChEMBL, Guide to Pharmacology, and PubChem), we developed a ligand search functionality. This approach allows for searching ligands using information from different sources of information, such as ligand names, database identifiers matched via UniChem (20), ligand InChIKeys (calculated with RDKit, http://www.rdkit.org), CAS numbers via the Entrez E-utilities (21), UniProt accession numbers (2) and sequences.

### Building an endogenous ligand browser

Endogenous ligand bioactivities and associated references were downloaded from the Guide to Pharmacology (https://www.guidetopharmacology.org/download.jsp). These data contain a ‘principal’ and ‘secondary’ classification of many, but not all, endogenous ligands that is important in many scientific studies, for example when selecting a reference ligand for physiology-biased signaling (22). For receptors lacking this classification and having only a single endogenous ligand we assigned the ‘principal’ category. To estimate the physiologically most important ligand for receptors with multiple unclassified ligands, we calculated a potency ranking for all endogenous ligands of each GPCR. This calculation was performed based on the mean pEC_50_, and if not available pK_i_, across studies.

## CURRENT DATA, ANALYSIS TOOLS AND EXPERIMENT DESIGN TOOLS

**Figure 1.**
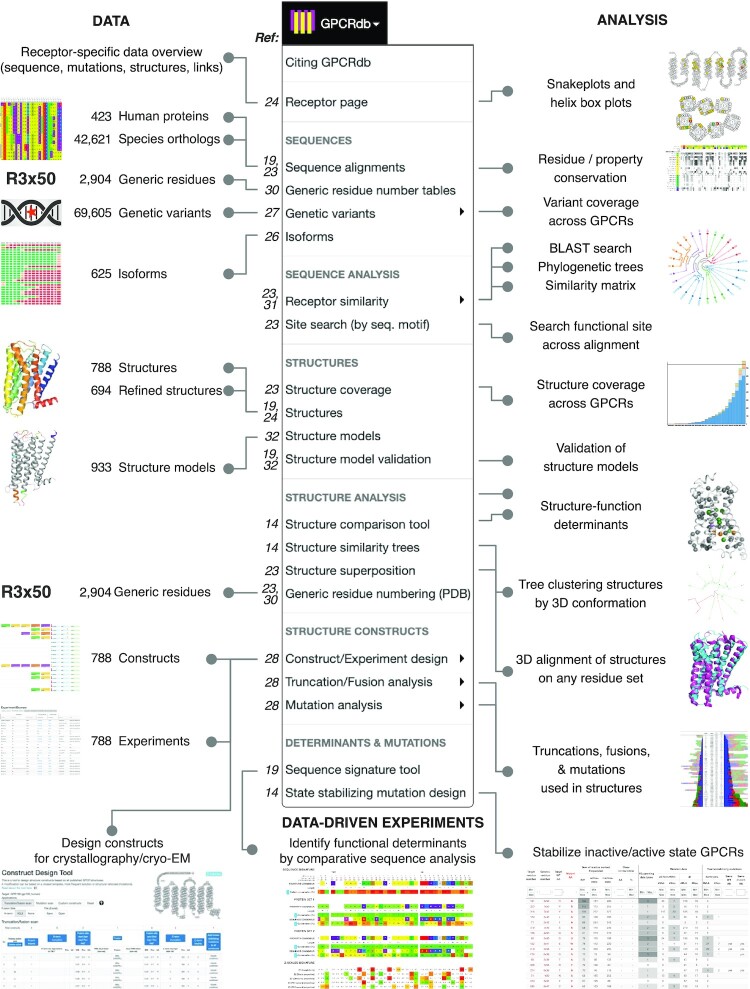
GPCR data, analysis resources and data-driven experiment tools in the GPCRdb 2023 release. The numbers to the left of each menu item are publication references with more information (including 30–32).

**Figure 2.**
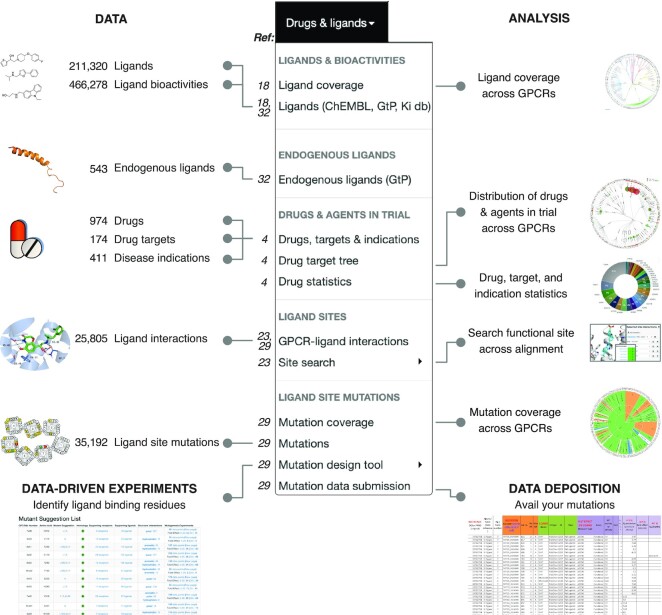
Drug/ligand data, analysis, data-driven experiment tool, and data deposition in the GPCRdb 2023 release. The numbers to the left of each menu item are publication references with more information.

Given that this is our fifth publication of a major GPCRdb release (18,19,23,24), and additional resources have published (14,25) after the latest publication (19), we provide an overview of the current data, analysis, data-driven experiment design, and data deposition in Figures 1 and 2. Data: The updated data types span receptor sequences, isoforms (26), genetic variants (27), structures, drugs and mutations used to pinpoint ligand binding residues or (thermo-)stabilize structures (28). The sequences and generic residue numbers were recently expanded to cover the Class D of Ste2 fungal pheromone receptors (25). For refined structures, structure models, and ligands (endogenous and surrogate) we describe major updates of data or functionality in the following sections. Analysis: The analysis tools cover all of the sequence, structural and ligand/drug data spaces. Recently, we added an online GPCR structure analysis platform featuring a tool to identify functional determinants from comparative structure analysis, and trees clustering receptors by their 3D conformation (14). Data-driven experiment design tools: The current set of data-driven tools serve to (i) design constructs for crystallography/cryo-EM studies (28), (ii) identify functional determinants from comparative sequence analysis, (iii) provide mutations stabilizing inactive/active states, and mutate ligand binding sites (29).

## STRUCTURES AND MODELS

### Structures and refined structures

GPCRdb makes monthly updates of GPCR structures from the Protein Data Bank (PDB) (33) that are complemented with manual annotation of additional information about the receptors, endogenous ligands, bound ligands, bound transducer proteins, and auxiliary proteins. This allows the *Structures* page to aid structure browsing and selection based on e.g. receptor classes, ligand, or structure properties, such as resolution. Since the last GPCRdb update publication (19), the number of annotated structures has increased from 488 to 788. Structures are also offered in a refined version wherein mutated residues are reverted to wildtype. Furthermore, missing regions are modelled in using AlphaFold models as swap-in templates for the missing coordinates.

### State-specific structure models using AlphaFold-Multistate

**Figure 3.**
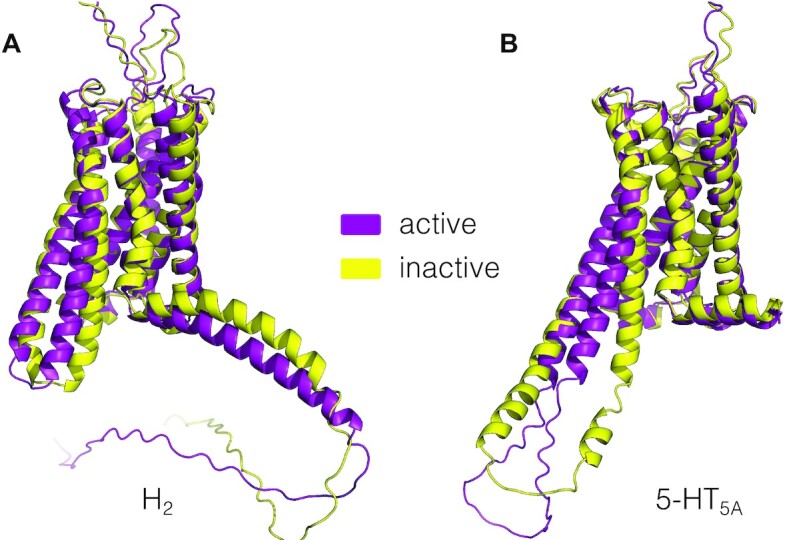
Structure models of the histamine H_2_ and serotonin 5-HT_5A_ receptors in both the active and inactive states built with AlphaFold-Multistate.

This release contains 844 inactive or active state GPCR structure models built using AlphaFold-Multistate (exemplified in Figure 3). The models can be downloaded from the *Structure models* page with or without loop and termini segments. In a model details page, users can view the 3D structure models color-coded by the AlphaFold confidence scores. All are full length models (include loops and termini) of wildtype sequence, except for AGRV1 which had to be reduced due to its extraordinary length of 6,306 amino acids. This includes 41 new and 71 longer receptors (82 and 142 models, respectively) compared to the AlphaFold-Multistate archive, and 5 receptors (10 models) that are not available from AlphaFold Protein Structure Database (12) website. To provide an initial validation, we calculated root-mean-square (RMSD) values for five target structures that were published after our models were built. The validation is available in the *Structure model validation* page and yielded an average RMSD of 1.6 Å for the backbone of transmembrane domain. This supports the use of AlphaFold-Multistate for the specific modelling of activation state of GPCRs.

The models will be updated twice a year including newly published structure templates and their determined activation state, thereby constantly improving the quality of the provided receptor models. In some cases, AlphaFold models do not represent a fully inactive or active state because there are no closely related structural templates. We will filter these models and sample more receptor conformations and explore rotamer optimization to improve docking of receptor ligands.

## LIGANDS

### Ligands and their coverage across GPCR targets

The GPCRdb ligand resource has been updated to incorporate the most recent version of ChEMBL (16) and add ligands from the Guide to Pharmacology (15) and PDSP K_i_ (https://pdsp.unc.edu/databases/kidb.php) databases. It currently contains 211 320 ligands and 466 278 binding affinity or potency values (6% (>20% if considering duplicates) and a 19% increase, respectively compared to GPCRdb's 2021 publication). The coverage of ligands across the receptors in the different human GPCR classes is A: 236 (75%), B1: 15 (100%), B2: 5 (15%), C: 12 (55%), F: 8 (73%) and T2: 25 (100%). Furthermore, the average number of ligands per receptor in each GPCR class is A: 612, B1: 400, C: 372, F: 90 and T2: 19.

### Ligand browser

The receptor target selection page has added selection of receptors that are drug targets through two new columns ‘Target of an approved drug’, and ‘Target in clinical trials’, as defined in DrugBank, and DrugCentral. After selecting the receptor target, users can choose between two ligand bioactivity browsers. The ‘Compact (1 row/ligand)’ browser collates all binding or functional bioactivities of a given ligand and source database on one row by calculating minimum, average and maximum activity across studies. The ‘Extended (1 row/activity)’ browser instead lists the specific binding affinity or potency value of each study. This GPCRdb release has restructured potency and affinity data into separate tabs. It has also added fold selectivity values, along with the underlying number of experiments, allowing ligand selection based on their selectivity for the target of interest relative to all other stored GPCR targets. Both browsers also present information about vendors from which one can purchase the given ligand along with key physicochemical descriptors (18).

### Ligand info page

**Figure 4.**
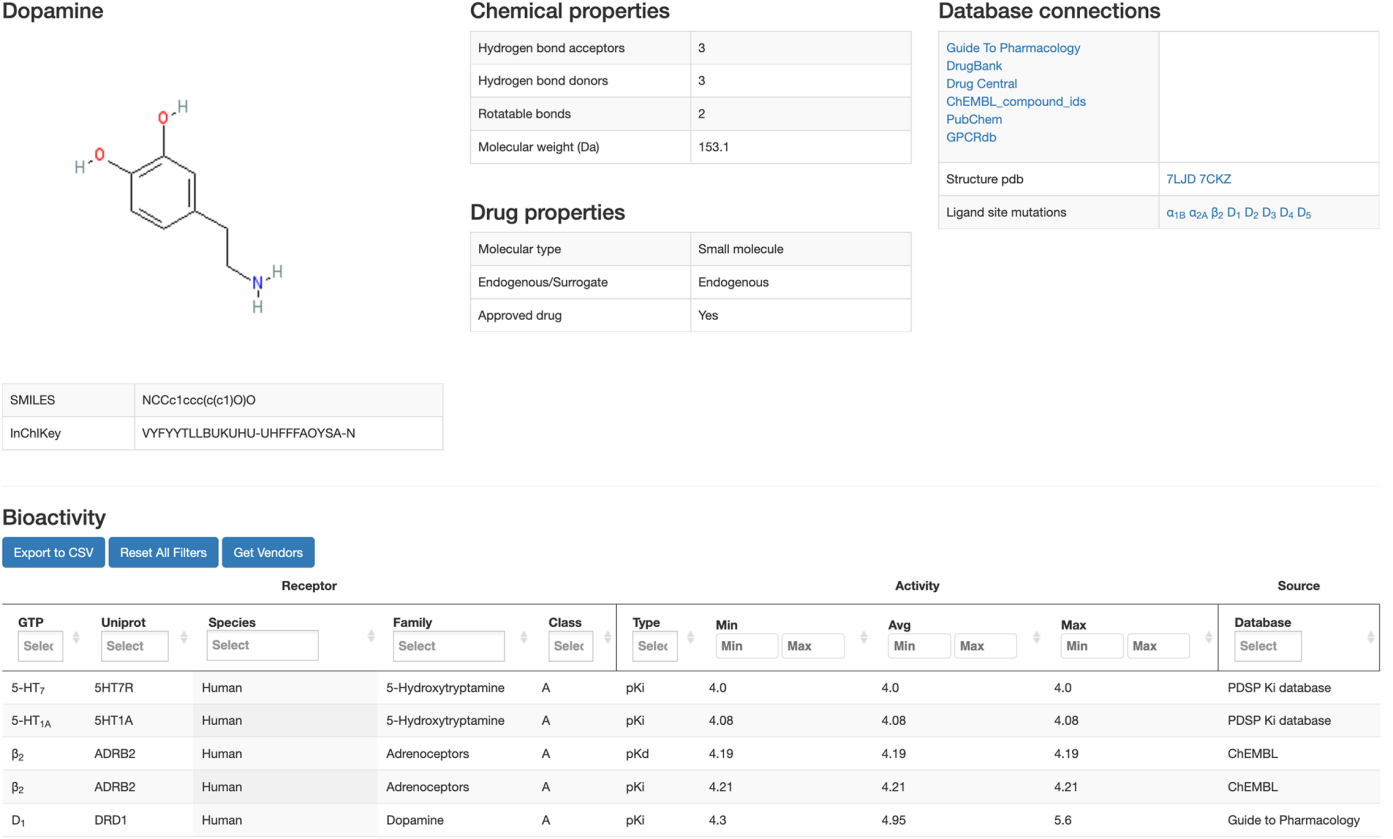
Ligand info page. The Ligands subsection of GPCRdb has been extended to 211 320 ligands and 466 278 binding affinity or potency values from the ChEMBL (16), Guide to Pharmacology (15) or PDSP K_i_ databases (https://pdsp.unc.edu/databases/kidb.php). The new ligand info page shows ligand structure, names, chemical properties, molecule type, drug status, and endogenous/surrogate status, and database links. When available, GPCR-ligand crystal/cryo-EM structure complexes and mutations affecting ligand activity are also shown. Bioactivities across receptor targets are shown in the browser at the bottom.

This GPCRdb release contains a new page for individual ligands and its targets (Figure 4). This can be accessed directly by a ligand query or from the results page of the above ligand browser. The top of the ligand info page displays information about ligand structure (2D image, SMILES, InChI key), names (common, and chemical names, and aliases), physicochemical properties (molecular weight, logP and counts for hydrogen bond acceptors/donors, and rotatable bonds), molecule type (small molecule/peptide/protein, drug status and endogenous/surrogate), and database links (internal and external). For ligands that have this information, two additional boxes provide GPCR-ligand crystal/cryo-EM structure complexes and mutations affecting ligand activity. The bottom of the page shows bioactivities for the given ligand across receptor targets. The bioactivity browser allows filtering by the receptor classification, bioactivity, and source database. The information on endogenous ligands and target FDA approval status have been derived from Guide to Pharmacology (15) and DrugBank (34) databases, respectively.

### Endogenous ligand browser

**Figure 5.**
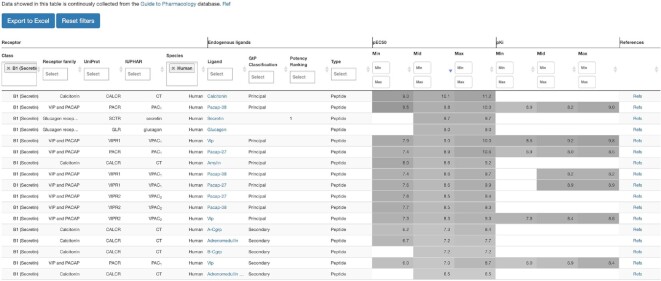
The endogenous ligand resource offers browsing across both multiple ligand and receptors. Four sections present (i) receptors (names and classification), (ii) endogenous ligands (name with link to detailed ligand info, ligand type (small molecule, peptide, protein), classification into principal, secondary or none categories, and potency rank), (iii) bioactivities (pEC_50_ and pK_i_ minimum, average, and maximum values across studies) and (iv) references (publication link). All endogenous ligands and their primary/secondary classification were derived from the Guide to Pharmacology database (15).

The endogenous ligand-GPCR system spans different relationships ranging (ligand:receptor) 1:1, 1:many, many:1 and many:many relationships (3). To facilitate browsing across either ligands or receptors, we developed an endogenous ligand browser (Figure 5). This browser contains data for 543 distinct endogenous ligands for 253 human GPCRs, and 157 mouse, rat, or guinea pig receptors. For each receptor, alternative endogenous ligands are classified as principal or secondary, as defined by the nomenclature committee of the International Union of Basic and Clinical Pharmacology (https://www.guidetopharmacology.org/nomenclature.jsp) and have an additional ranking by potency. For ligand-receptor pairs with multiple potency (pEC_50_) or affinity (pKi) values, the browser provides minimum, mean and maximum values, with grayscale background aiding comparison. Finally, the browser contains information about the ligand type, ligand name with a direct link to the ligand info page in GPCRdb, receptor information as family, species, IUPHAR and UniProt name and a popup showing the original references for bioactivities.

## DATA AVAILABILITY

GPCRdb is available at https://gpcrdb.org and can also be accessed via a RESTful API, which complies with the OpenAPI specification using Swagger (code examples are available at https://docs.gpcrdb.org/web_services.html). The source code, the underlying data and a virtual machine configuration are all available in the repositories at https://github.com/protwis/.

## Notes

Present address: Alibek Mamyrbekov, Trial Data Management, Novo Nordisk A/S, Vandtårnsvej 108, 2860 Søborg, Denmark.
